# Low-dose IL-2 improved clinical symptoms by restoring reduced regulatory T cells in patients with refractory rheumatoid arthritis: A randomized controlled trial

**DOI:** 10.3389/fimmu.2022.947341

**Published:** 2022-11-29

**Authors:** Jia Wang, Sheng-Xiao Zhang, Jia-Song Chang, Ting Cheng, Xiao-Jing Jiang, Qin-Yi Su, Jia-Qi Zhang, Jing Luo, Xiao-Feng Li

**Affiliations:** ^1^ Department of Rheumatology, The Second Hospital of Shanxi Medical University, Taiyuan, China; ^2^ Key Laboratory of Cellular Physiology at Shanxi Medical University, Ministry of Education, Taiyuan, China; ^3^ Department of Physiology, Shanxi Medical University, Taiyuan, China

**Keywords:** rheumatoid arthritis, refractory, interleukin-2, Th17, CD4 Tregs, immunoregulation

## Abstract

**Background:**

Regulatory T cells (Tregs) have been found to play crucial roles in immune tolerance. However, the status of Tregs in refractory rheumatoid arthritis (RA) is still unclear. Moreover, low-dose interleukin-2 (IL-2) has been reported to selectively promote the expansion of Tregs. This study investigated the status of CD4^+^ Tregs and low-dose IL-2 therapy in patients with refractory RA.

**Methods:**

The absolute number of CD4^+^CD25^+^FOXP3^+^ Treg (CD4 Treg), CD4^+^IL17^+^ T (Th17), and other subsets in peripheral blood (PB) from 41 patients with refractory RA and 40 healthy donors was characterized by flow cytometry combined with an internal microsphere counting standard. Twenty-six patients with refractory RA were treated with daily subcutaneous injections of 0.5 million IU of human IL-2 for five consecutive days. Then, its effects on CD4 Treg and Th17 cells in PB were analyzed.

**Results:**

A decrease in the absolute number of PB CD4 Tregs rather than the increase in the number of Th17 was found to contribute to an imbalance between Th17 and CD4 Tregs in these patients, suggesting an essential role of CD4 Tregs in sustained high disease activity. Low-dose IL-2 selectively increased the number of CD4 Tregs and rebalanced the ratio of Th17 and CD4 Tregs, leading to increased clinical symptom remission without the observed side effects.

**Conclusions:**

An absolute decrease of PB CD4 Tregs in patients with refractory RA was associated with continuing disease activation but not the increase of Th17 cells. Low-dose IL-2, a potential therapeutic candidate, restored decreased CD4 Tregs and promoted the rapid remission of patients with refractory RA without overtreatment and the observed side effects.

**Clinical trial registration:**

http://www.chictr.org.cn/showproj.aspx?proj=13909, identifier ChiCTR-INR-16009546.

## Introduction

Rheumatoid arthritis (RA) is a chronic inflammatory joint disease that potentially leads to cartilage, bone damage, and disability. Treatment algorithms involve measuring disease activity with composite indices, applying a treatment-to-target strategy, and using conventional, biological, and new non-biological disease-modifying antirheumatic drugs (DMARDs) (1). Though these strategies have good efficacy, up to 40% of patients did not benefit from these therapies due to lack of efficacy or development of resistance (2, 3) or treatment-related adverse events (4, 5). Adequate control of disease activity in RA is achieved in only approximately one-third of all RA patients with previous treatments (6, 7). Accordingly, new therapies are urgently required for refractory RA exposed to multiple DMARDs without necessarily benefitting from them (8).

Effector T cells, such as the Th17 subset of CD4^+^ T cells producing interleukin-17 (IL-17), have been reported to play important roles in inflammation (9). Th17 cells mediate the inflammation process by stimulating the production of cytokines and chemokines (10). IL-17 levels are extremely low or undetectable in normal human peripheral blood, while the levels are elevated in peripheral blood or synovial fluid in RA patients (11). The frequency of Th17 in peripheral blood was reported to increase in RA patients (12, 13), while the role of Th17 in refractory RA remains to be studied.

The pathogenesis of RA is further closely associated with the imbalance of pro-inflammatory Th17 cells and anti-inflammatory Treg cells (14). Treg cells are a distinct set of T cells responsible for suppressing autoreactive deleterious activities of effector T cells (15). Our previous study reported that reduced circulating Tregs (CD4^+^CD25^+^Foxp3^+^ Treg) might be involved in the pathogenesis and progression of RA (16). Although several studies (17) evaluated the proportion of Tregs in active RA and remission RA patients, the definition of Treg cells was different between these works. However, the status of Treg cells in refractory RA is still unclear.

IL-2, a cell growth factor, plays a critical role in regulating immune balance by providing a survival and proliferation signal for various types of T cells (18). However, high-dose IL-2 administration has been confirmed to enhance immune responses by activating effector T cells against cancer (19, 20), while low-dose IL-2 was evidenced to mainly stimulate Treg cell survival and expansion and thereby control autoimmunity and inflammation. Low-dose IL-2 at 0.5 million IU was widely used in the treatment of several autoimmune diseases, such as systemic lupus erythematosus (20, 21), psoriatic arthritis (22), and RA (16), but not refractory RA. However, due to the lack of evidence of decreased Treg cells in patients with refractory RA, the immune regulation therapy of IL-2 in these patients has not been examined.

The present study compared the status of PB lymphocyte and CD4^+^ T cell subsets of refractory RA with healthy donors. Furthermore, we explored whether low-dose IL-2 could effectively induce the remission of refractory RA by upregulating Treg cells.

## Materials and methods

### Participants

A total of 41 patients with RA and 40 healthy individuals were enrolled in the study. The patients fulfilled the 2010 rheumatoid arthritis classification criteria (23). The main characteristics of RA patients and age- and sex-matched healthy donors are shown in **Supplemental Table 1**. These patients between the age of 18 and 65 had severely active rheumatoid arthritis [≥8 tender joints of 28 joints examined, ≥3 swollen joints of 28 joints examined, morning stiffness lasting longer than 60 min, a serum C-reactive protein (CRP) level that is at least 1.5 times of the upper limit or an erythrocyte sedimentation rate (ESR) that is at least 28 mm per hour, and DAS28 ≥5.1]. At study entry, patients must have been regularly taking one or more conventional DMARDs for at least the preceding 6 months but without disease activity remission. Patients must also have a negative pregnancy test and agree to use effective contraception during the study and for at least 6 months after stopping study treatment. They were able to comply with scheduled visits, treatment plans, laboratory tests, and other study procedures. The patients were excluded if they had a history of malignancy, were suffering from malignant disease within 5 years prior to study entry, or had a recent clinically significant infection. Patients in a pregnancy test who disagreed with using effective contraception during the study or at least 6 months after stopping study treatment were also excluded.

### Study design and oversight

The patients were divided randomly into two groups: the non-IL-2 group (*n* = 15), who were still given conventional glucocorticoid and DMARD treatment, and the IL-2 group (*n* = 26), who were not only given the same conventional treatment but also injected subcutaneously IL-2 at 0.5 million IU per day for five consecutive days (24). Before and after the treatment, the patient’s peripheral lymphocyte subpopulation and CD4^+^ T subgroups were measured by flow cytometry. The absolute numbers of those cells were compared between the non-IL-2 and IL-2 groups before and after treatment.

### Flow cytometric analysis

#### Absolute number by adding an internal microsphere counting standard

In this study, BD Trucount™ tubes with the lyophilized pellet of a known number of fluorescent beads were used for determining the absolute counts of T, B, NK, CD8^+^ T, and total CD4^+^ T cells in PB and calculating the percentage and the absolute number of CD4^+^ T subsets, especially that of Th17 and CD4 Tregs.

#### Analysis of T, B, CD4^+^ T, and CD8^+^ T cells in PB

For the analysis of T, B, CD4^+^ T, and CD8^+^ T cells, 50 µl of fully blending whole anticoagulant blood was reversely added into two Trucount tubes carefully without touching the standard beads that are in the bottom of the tube. Both tubes were added with 20 μl of anti-CD3FITC/CD8PE/CD45PercP/CD4APC antibodies and anti-CD3FITC/CD16+56PE/CD45PercP/CD19APC antibodies, respectively, totally mixed by a vortex blender. Cells were stained with antibodies for CD3^+^ (T), CD3^+^/CD19^+^ (B), CD3^+^/CD4^+^ (CD4^+^ T), CD3^+^/CD8^+^ (CD8^+^ T), and CD3^-^CD16^+^CD56^+^ (NK). All antibodies were purchased from BD (USA) (**Supplemental Table 2**).

#### Analysis of Th1, Th2, Th17, and CD4 Tregs in PB

For the analysis of Th1, Th2, and Th17 cells, cells in 1 ml of heparin-anticoagulated venous blood were stimulated for 5 h with 10 μl of PMA, 10 μl of ionomycin (final concentration was 750 ng/ml), and 1 μl of GolgiStop, respectively, in a 37°C incubator. Stimulated cells (80 μl) were taken for surface staining, and the cells were fixed and permeabilized using fixation/permeabilization reagent (BD, USA) and then stained with anti-IFN-γ-APC (for Th1), anti-IL-4-PE (for Th2), and anti-IL-17A-PE (for Th17) monoclonal (**Supplemental Table 2**).

For the analysis of CD4 Tregs, cells in 80 μl of heparin-anticoagulated venous blood were aliquoted into tubes without PMA and ionomycin stimulation and surface-labeled with anti-CD4-FITC and anti-CD25-APC followed by fixation, permeabilization, and intracellular staining with anti-FoxP3-PE (all from BD, USA). The labeled cells were washed and analyzed with a FACSCalibur flow cytometer (Becton-Dickinson) using the CellQuest software (BD, USA). At least 5,000 to 10,000 cells were collected to calculate the percentage of these CD4^+^ T subsets, and the total number of CD4^+^ T cells was calculated using the internal microsphere counting standard.

In this study, we used an equation to calculate the absolute number of CD4^+^ T subsets: the absolute number of CD4^+^ T subsets = the percentage of each CD4^+^ T subset * the absolute number of total CD4^+^ T cells.

### Efficacy measurement of IL-2 treatment

At the primary endpoint of IL-2 treatment, the number of tender swollen joints and disease activity were assessed using the 28-joint Disease Activity Score (based on the erythrocyte sedimentation rate, ESR). The primary comparison was between the group receiving conventional treatment and the group receiving IL-2 at a dose of 0.5 million IU for a 5-day course. Secondary measures included the level of high-sensitivity CRP and ESR.

### Safety measurement of IL-2 treatment

Clinical laboratory tests, measurement of vital signs, and other safety assessments were performed at scheduled visits. The incidence and severity of all adverse events were recorded. The blood routine and the liver and renal function were detected before and after the treatment.

### Statistical analysis

The demographic parameters of the healthy individuals and RA patients were compared using an unpaired *t*-test for parametric data (age, PB lymphocyte subpopulations, and CD4^+^ T subsets) and the *χ* ^2^ test for proportions (sex). Blood routine, liver and renal function, and disease activity were measured in two different treatment groups before and after the treatment. The paired *t*-test was used to analyze the influence of IL-2 on peripheral lymphocytes and CD4^+^ T subsets in patients receiving IL-2 subcutaneous injection. Correlation analysis was performed using the Spearman correlation coefficient. All *p*-values reported herein are two-tailed. A *p*-value <0.05 was taken as statistical significance. The software used for the statistical analysis was SPSS 13.0 and GraphPad Prism 5.0.

## Results

### Refractory RA patients had an imbalanced Th17/CD4 Treg homeostasis by decreasing CD4 Tregs, rather than increasing Th17 cells

CD4 Tregs and Th17 cells were analyzed by flow cytometry. Though Th17 cells are considered to be a leading actor in the autoimmunity scenario, our results showed that the number of peripheral Th17 had no significant difference between patients with refractory RA and healthy donors [9.0 (5.1–16.8) vs. 7.4 (4.3–10.8), *p* = 0.132]. Interestingly, the absolute number of CD4 Tregs was significantly lower in RA patients compared with that in healthy donors [19.5 (12.2–28.9) vs. 35.5 (24.6–46.7), *p* < 0.001]. Accordingly, the ratio between Th17 and CD4 Treg in RA patients was significantly elevated as compared with that in the control group [0.59 (0.24–1.07) vs. 0.20 (0.16–0.31), *p* < 0.001]. The absolute number of CD4 Tregs was much lower [19.5 (12.2–28.9)] in those RA patients, especially for those who received long-term immunosuppression by conventional glucocorticoid, NSAID, or DMARD treatment (data not shown). No significant difference was observed in terms of age (55.1 ± 13.83 vs. 50.9 ± 9.49, *p* = 0.111) and sex (*p* = 0.809) between RA patients and healthy donors. Except for Tregs and B cells, all the other populations also showed no significant changes (**Supplemental Table 1**).

### The reduction of CD4 Treg cell numbers was closely correlated with higher disease activity

As shown in **Supplemental Table 3**, the CD4 Treg values were found to be slightly negatively correlated with DAS28 (*r* = −0.625, *p* < 0.001), ESR (*r* = −0.408, *p* = 0.001), CRP (*r* = −0.344, *p* = 0.009), number of joint pain (*r* = −0.639, *p* < 0.001), and number of joint swollen (*r* = −0.538, *p* < 0.001). The ratio of Th17 cell to CD4 Treg cell was found to be correlated with DAS28 (*r* = 0.350, *p* = 0.004), number of joint pain (*r* = 0.393, *p* = 0.001), and number of joint swollen (*r* = 0.407, *p* = 0.001). These data point to a relevant contribution of the deficiency of CD4 Tregs and an imbalanced Th17/CD4 Treg homeostasis to disease-continuing activity.

### Low-dose IL-2 selectively reversed CD4 Treg defects and expanded PB CD4 Tregs in patients with RA

**Figure 1:**
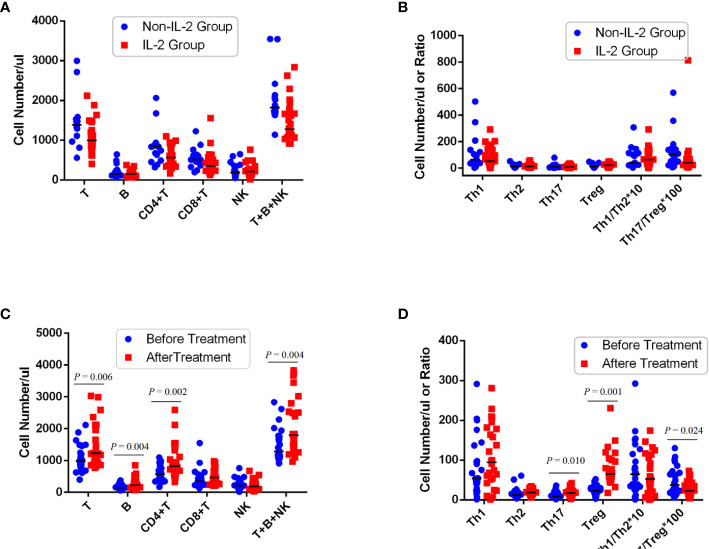
Peripheral CD4 Treg cells markedly decreased in refractory RA patients and restored after IL-2 treatment. Absolute number of lymphocyte subpopulations **(A)** or CD4^+^ T subsets **(B)** in PB of RA patients treated with or without IL-2 before this therapy. Status of peripheral lymphocyte subpopulations **(C)** or CD4^+^ T subsets **(D)** in patients with IL-2 before and after treatment. Absolute values of the cells (median, quartile range) are shown.

**Figure 2:**
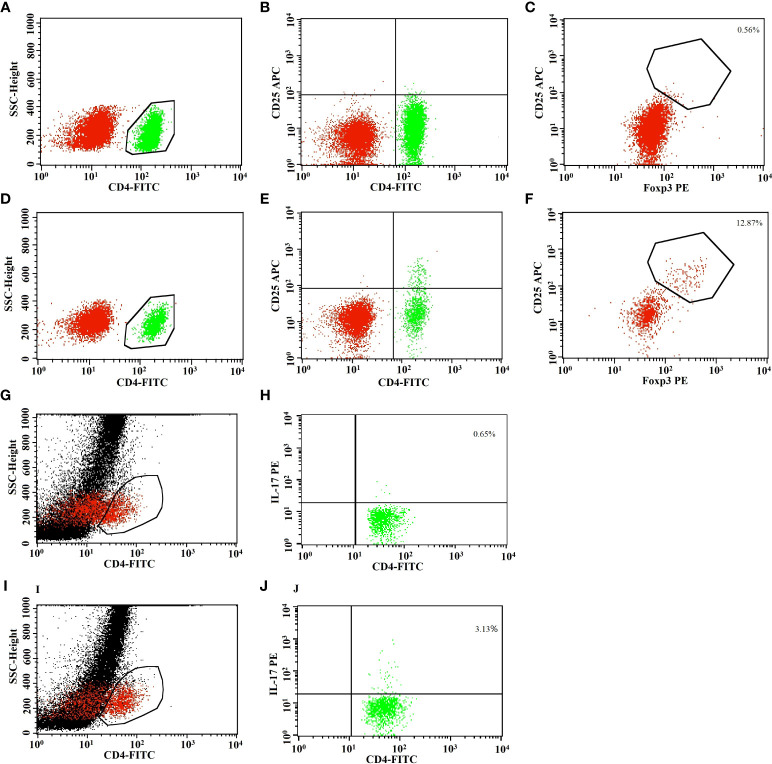
Representative flow cytometric analysis showed an obvious increase of CD4 Treg cells after low-dose IL-2 treatment. Representative flow cytometric analysis of peripheral CD4^+^ T, CD4^+^CD25^+^ T, CD4 Tregs (CD4^+^CD25^+^FOXP3^+^ T), and Th17 cells (CD4^+^IL17^+^ T) was shown in refractory RA after low-dose IL-2 treatment. The results revealed the increase of CD4^+^ T cells **(A, D, G, I)**, CD4^+^CD25^+^ T cells **(B, E)**, CD4 Tregs **(C, F)**, or Th17 cells **(H, J)** in CD4^+^ T-cell population after IL-2 treatment **(D–F, I, J)** compared to before **(A–C, G, H)**.

Low-dose IL-2 can selectively expand CD4 Tregs *in vivo* (25). Therefore, we next evaluated whether the observed CD4 Treg defects in patients with refractory RA could be restored *in vivo* by subcutaneous injection of IL-2. Twenty-six of 41 patients received recombinant human IL-2 (aldesleukin) at a dose of 0.5 million IU for five consecutive days. Complete whole blood from patients was repetitively collected on days 1 and 5 of IL-2 stimulation. Before IL-2 treatment, there were no significant differences in the numbers of CD4^+^ T-cell subsets between the IL-2 group and the non-IL-2 group (**Figures 1A, B** and **Supplemental Table 4**). In contrast, the percentage of CD4 Treg cells was higher in the IL-2 group (**Supplemental Figure 1**). After IL-2 treatment (**Figures 1C, D** and **Table 1**), compared with before treatment as well as with non-IL-2 control, there was a significant increase in T cells [990 (799, 1,301) vs. 1,227 (954, 2,026) cells/µl, *p* = 0.015], B cells [144 (80, 213) vs. 218 (149, 445) cells/µl, *p* = 0.005], CD4^+^ T cells [564 (417, 832) vs. 823 (597, 1,334) cells/µl, *p* = 0.003], and total lymphocyte cells [1,282 (1,109, 1,761) vs. 1,798 (1,414, 2,600) cells/µl, *p* = 0.006)]. The absolute count and percentage of CD4 Tregs [23 (14, 30) vs. 65 (48, 102) cells/µl and 3.7% (2.7%, 5.1%) vs. 8.5% (6.2%, 10.7%), *p* < 0.001] were dramatically elevated by 3-fold (**Table 1**; **Supplemental Figure 2**). There was also an increase in the absolute number of Th17 cells [9 (4, 16) vs. 17 (9, 29) cells/µl, *p* = 0.008]. However, it is noteworthy that the CD4 Tregs were increased much more dramatically than the Th17 cells (**Figures 1**, **2**), leading to a decrease in their ratio [0.40 (0.23, 0.76) vs. 0.23 (0.12, 0.44), *p* = 0.010].

### IL-2 rapidly induced patients’ clinical remission

Before the treatment, there was no difference in disease activity measures between the IL-2 group and the non-IL-2 group (*p *> 0.05) (**Table 2**). But after IL-2 subcutaneous injection combined with the same conventional antirheumatic drugs, there was a significant decrease in DAS28 score (3.60 ± 0.96 vs. 2.85 ± 0.67, *p* = 0.005), 28 tender joint count (3.73 ± 2.79 vs. 0.94 ± 1.00, *p* = 0.001), and swollen joint count (1.40 ± 1.64 vs. 0.42 ± 0.70, *p* = 0.011) in the IL-2 group compared with the non-IL-2-treated individuals (**Table 3**). The above results suggest that although the numbers of T, B, CD4^+^ T, and Th17 cells were increased, as shown in **Table 1**, these changes contribute to tipping the immune status toward balance rather than inflammation.

**Table 1:** Comparison of PB lymphocyte subsets and CD4^+^ T subsets in IL-2-treated patients before and after IL-2 treatment (*N* = 26).

Variables	Before treatment (*N* = 26)	After treatment (*N* = 26)	*p*-value
PB lymphocyte subsets (cells/µl or ratio)	Median (quartile range)	Median (quartile range)	
T	990 (799, 1,301)	1,227 (954, 2,026)	0.015*
B	144 (80, 213)	218 (149, 445)	0.005**
CD4^+^ T	564 (417, 832)	823 (597, 1,334)	0.003**
CD8^+^ T	354 (231, 452)	463 (266, 713)	0.077
CD4^+^ T/CD8^+^ T	1.48 (1.09, 2.30)	2.21 (1.26, 2.73)	0.280
NK	212 (125, 270)	186 (99, 274)	0.654
T+B+NK	1,282 (1,109, 1,761)	1,798 (1,414, 2,600)	0.006**
CD4^+^ T subgroups (cells/µl or ratio)			
Th1	54 (36, 139)	95 (50, 179)	0.200
Th2	13 (6, 18)	19 (13, 25)	0.015*
Th17	9 (4, 16)	17 (9, 29)	0.008**
Tregs	23 (14, 30)	65 (48, 102)	<0.001***
Th1/Th2	6.50 (2.85, 11.83)	5.24 (2.60, 10.78)	0.583
Th17/Tregs	0.40 (0.23, 0.76)	0.23 (0.12, 0.44)	0.010*

IL-2 significantly increased the number of T, B, CD4^+^ T, CD8^+^ T, Th17, and CD4 Treg cells (p < 0.05). The ratio of Th17/Treg decreased after IL-2 treatment (p < 0.05).

PB, peripheral blood; NK, natural killer; Th, helper T; Tregs, regulatory T cells.

*p < 0.05; **p < 0.01; ***p < 0.001.

**Table 2:** Baseline characteristics and disease activity measures of patients before IL-2 treatment (*N* = 41).

Variables	Non-IL-2 group (*N* = 15)	IL-2 group (*N* = 26)	*p*-value
Female/male	12/3	15/11	0.212
Mean age, years, mean ± SD	54.31 ± 17.85	55.65 ± 11.30	0.917
Duration of RA, month, mean ± SD	79.19 ± 71.60	136.81 ± 138.11	0.429
Prednisone dose, mg/day, median (quartile range)	15 (10, 15)	10 (7.9, 15)	0.192
DMARDs	Yes	Yes	
NSAIDs	Yes	Yes	
Blood routine	Mean ± SD	Mean ± SD	
RBC, ×10^12^/L	4.27 ± 0.47	4.27 ± 0.48	0.981
Hemoglobin, g/L	123.97 ± 17.75	133.8 ± 142.46	0.556
Platelet, ×10^9^/L	307.35 ± 102.16	287.19 ± 94.36	0.526
Lymphocyte, ×10^9^/L	1.72 ± 0.58	1.50 ± 0.51	0.220
Liver function			
Alanine aminotransferase, U/L	16.75 ± 6.78	17.26 ± 10.78	0.871
Aspartate aminotransferase, U/L	18.27 ± 6.00	18.23 ± 7.24	0.984
Renal function			
Blood urea nitrogen, mmol/L	5.28 ± 1.38	4.88 ± 1.52	0.403
Creatinine, mmol/L	53.93 ± 17.72	54.32 ± 15.48	0.943
Disease activity measures			
DAS28-ESR	5.50 ± 1.35	5.31 ± 1.18	0.649
Erythrocyte sedimentation rate, mm/h	60.90 ± 32.57	53.92 ± 35.74	0.536
C-reactive protein, mg/L	57.18 ± 78.34	33.15 ± 37.84	0.192
28 tender joint count	10.30 ± 8.90	10.54 ± 8.79	0.943
28 swollen joint count	5.30 ± 6.00	4.15 ± 5.03	0.504

Patients were randomly divided into two groups. There was no difference in sex, age, duration, and conventional drug treatments between the two groups (p > 0.05). Liver and renal functions had no significant differences, too (p > 0.05). No significant differences in disease activity measures such as DAS28, ESR, CRP, and 28 tender and swollen joint counts between the two groups (p > 0.05).

DMARDs, disease-modifying antirheumatic drug; NSAIDs: non-steroidal anti-inflammatory drugs.

**Table 3:** Baseline characteristics and disease activity measures of patients after the treatment (*N* = 41).

Variables	Non-IL-2 group (*N* = 15)	IL-2 group (*N* = 26)	*p*-value
Disease activity measures	Mean ± SD	Mean ± SD	
DAS28-ESR	3.60 ± 0.96	2.85 ± 0.67	0.005**
ESR, mm/h	22.07 ± 17.92	22.77 ± 18.69	0.907
28 swollen joint count	3.73 ± 2.79	0.94 ± 1.00	0.001**
28 tender joint count	1.40 ± 1.64	0.42 ± 0.70	0.011*
Blood routing			
WBC, ×10^9^/L	11.82 ± 4.19	8.55 ± 3.14	0.007**
RBC, ×10^12^/L	4.14 ± 0.39	4.24 ± 0.57	0.551
Hemoglobin, g/L	118.87 ± 16.81	124.65 ± 19.45	0.342
Platelet, ×10^9^/L	273.33 ± 73.22	256.42 ± 84.51	0.522
Lymphocyte, ×10^9^/L	2.57 ± 1.54	2.18 ± 0.94	0.311
Liver function			
Alanine aminotransferase, U/L	28.91 ± 14.07	29.14 ± 19.41	0.973
Aspartate aminotransferase, U/L	17.65 ± 7.73	17.90 ± 8.64	0.936
Renal function			
Blood urea nitrogen, mmol/L	6.48 ± 1.11	5.79 ± 1.56	0.329
Creatinine, mmol/L	56.50 ± 5.09	60.05 ± 14.37	0.563

DAS28 and tender and swollen joint counts were much improved in the IL-2 group compared with the non-IL-2 group (p < 0.05). No difference was observed in blood routine and liver and renal functions after treatment compared with the IL-2 and non-IL-2 groups (p > 0.05).

DAS, disease activity score; ESR, erythrocyte sedimentation rate; WBC, white blood cell; RBC, red blood cell.

*p < 0.05; **p < 0.01.

### There was no severe adverse event observed after IL-2 treatment

The safety of this treatment is shown in **Tables 2**, **3**. There were no differences in blood routine, serum alanine transaminase (ALT), aspartate aminotransferase (AST), blood urea nitrogen (BUN), and serum creatinine (Cr) between the two groups before the treatment (*p *> 0.05) (**Table 2**). After the treatment, these measurements were also comparable in these two groups (*p *> 0.05) (**Table 3**). Except for mild reactions at the site of injection in some subjects (2 of 26 patients), no other side effects were observed.

## Discussion

The status of T-cell subsets in peripheral blood in patients with RA was controversial, especially that of Th17 and Treg cells (26). A meta-analysis of all 36 selected studies (17) revealed no significant difference in the proportion of the so-called Tregs between RA patients and control subjects. The inconsistent definitions of Tregs have been used in these studies, leading to different results. As a major CD4^+^ T-cell subset of Treg cells that express CD4 and FOXP3 (a transcription factor that plays a vital role in the function of Tregs) (27), we studied CD4 Tregs (CD4^+^CD25^+^FOXP3^+^). Our study firstly showed that the reduction of the absolute number or proportion of CD4 Tregs in PB was associated with the disease activity of refractory RA but not that of Th17 cells.

It is reported that Th17 cells and IL-17 levels are low or undetectable in regular human PB, while the levels are elevated in PB or synovial fluid in RA patients (12). CD4^+^ T-cell subsets in the CD4^+^ T lymphocyte population of PB include Th1, Th2, Treg, and Th17 cells. Generally, the proportions of these subsets in PB CD4^+^ T cells are used to quantify their levels. However, we found that the number of CD4 Tregs was almost 5-folds higher than that of Th17 in PB of healthy controls. Therefore, the number of CD4 Tregs greatly affects the percentage of Th17 cells in the PB CD4^+^ T population. In previous studies, the increased frequencies in Th17 cells may not be considered a change in Th17. On the contrary, it may suggest a decrease in the CD4 Treg cells. Perhaps only absolute numbers can precisely reflect the actual status of cells but not their frequency. Some flow cytometers cannot directly provide the cell concentration or total counts of cells in a sample. Absolute cell counts can be obtained by adding an internal microsphere-counting standard to the flow cytometric sample (single platform testing). The single-platform method is preferred as it is technically less complicated as it avoids interlaboratory variation and underestimations, making it more accurate than multiple-platform testing. In this study, BD Trucount™ tubes with the lyophilized pellet of a known number of fluorescent beads were used for determining the absolute counts of T, B, NK, and total CD4^+^ T cells in PB and then calculating the absolute number of CD4^+^ T subsets, especially that of Th17 and CD4 Tregs.

Our results showed that Th17 cells did not increase in patients with refractory RA compared with healthy controls. Therefore, the change in the Th17 number should not be the direct cause of refractory RA. Indeed, for these patients who received the long-term immunosuppressor treatment, the normal levels of Th17 cells might represent the treatment results (28–31). On the other hand, immunosuppressants also decrease the number or function of Tregs non-selectively (32, 33). These changes may aggravate the disturbance of immune balance. Therefore, disease activity relapses after drug withdrawal due to insufficient regulatory T cells, which might be an essential cause of refractory RA (34).

Moreover, long-term immunosuppressive therapies also lead to drug resistance (35, 36), which may be another cause of refractory RA. Therapeutic unresponsiveness may be caused by various mechanisms, such as the rapid degradation of corticosteroids (CCS), the release of neutralizing antibodies against biological agents such as infliximab, or other factors (37, 38). Moreover, long-term immunosuppressants downregulated the level of effector T cells, leading to a higher risk of infection and malignancy (39, 40).

Therefore, we propose an immunoregulation therapy to induce and reconstitute immune tolerance by low-dose IL-2 subcutaneous injection instead of simple immunosuppression. CD4 Tregs express high levels of CD25 (alpha-chain of the IL-2 receptor), so IL-2 was able to increase the level of Tregs (41). Our results demonstrated that low-dose IL-2 effectively increased many kinds of lymphocyte cells. The number of Th17 cells increased slightly, while that of CD4 Tregs soared dramatically. The increased CD4 Tregs contributed to tipping the immune balance and inhibiting inflammation for RA patients. Recent studies have reported the capacity for plasticity of the Th17 and Treg phenotypes. There is growing evidence that IL-17(+)/FoxP3(+) Treg clones also exhibit plasticity to secrete pro-inflammatory cytokines, such as IL-17A, in inflammatory conditions and retain their suppressive function (42, 43), which probably supports the finding regarding the increasing number of Th17 in the study.

These increased lymphocytes in PB may also be beneficial by relieving patients’ clinical symptoms without the expected adverse effects. Though no significant difference in ESR and CRP was observed between these groups for this short-time observation, there were substantial decreases in DAS28 and tender and swollen joint counts in the IL-2 group, compared with the non-IL-2-treated individuals who received almost the same conventional antirheumatic drugs after the treatment. Therefore, we confirm that low-dose IL-2 injection can help the remission of RA disease activity rapidly. As for the safety of IL-2 injection, no severe adverse event was observed except for mild reactions at the injection site.

However, we only observed this study for 1 week, and a long-term study is needed. According to the levels of Th17 and CD4 Tregs, we are trying to maintain the balance of immune function by low-dose IL-2 once a week. We are also trying other methods to maintain a long-term immune balance and induce and reconstitute immune tolerance for patients with immunodeficiency. In addition, this study also has some limitations, such as being a single-center study, having a small sample size, and implementing short-term monitoring. A multicenter study, a maximum sample, and long-term clinical studies are still extremely necessary.

In conclusion, the disease activity of refractory RA is associated with the absolute number of CD4 Tregs but not that of Th17 cells and other populations. Low-dose IL-2 can effectively upregulate the level of CD4 Treg as well as that of Th17 to some degree and maintain the balance of Th17 and CD4 Tregs. Subcutaneous injection of IL-2 combined with traditional antirheumatic drugs may help in the rapid remission of RA patients’ symptoms without overtreatment and the expected side effects. However, the long-term benefits of this immune regulation therapy need to be clarified by conducting further studies.

## Data availability statement

The original contributions presented in the study are included in the article/**Supplementary Material**. Further inquiries can be directed to the corresponding author.

## Ethics statement

This study was approved by the Ethics Committee of the Second Hospital of Shanxi Medical University (2016 KY-007). The patients/participants provided their written informed consent to participate in this study.

## Author contributions

Study design and manuscript writing: JW and S-XZ. Data extraction, quality assessment, analysis, and interpretation of data: JW, TC, J-SC, and X-JJ. All authors approved the final version to be published. X-FL had full access to all the data in the study and took responsibility for the integrity of the data and the accuracy of the data analysis.

## Funding

This work was supported by the National Natural Science Foundation of China (No. 82001740).

## Conflict of interest

The authors declare that the research was conducted in the absence of any commercial or financial relationships that could be construed as a potential conflict of interest.

## Publisher’s note

All claims expressed in this article are solely those of the authors and do not necessarily represent those of their affiliated organizations, or those of the publisher, the editors and the reviewers. Any product that may be evaluated in this article, or claim that may be made by its manufacturer, is not guaranteed or endorsed by the publisher.
