# Supplementary material for: Low-dose IL-2 improved clinical symptoms by restoring reduced regulatory T cells in patients with refractory rheumatoid arthritis: A randomized controlled trial
Source: Front Immunol. 2022 Nov 29;13:947341. doi: 10.3389/fimmu.2022.947341 (PMC9744779; doi:10.3389/fimmu.2022.947341)
Supplement: Supplementary file 1 [file Presentation_1.ppt]

## Slide 1
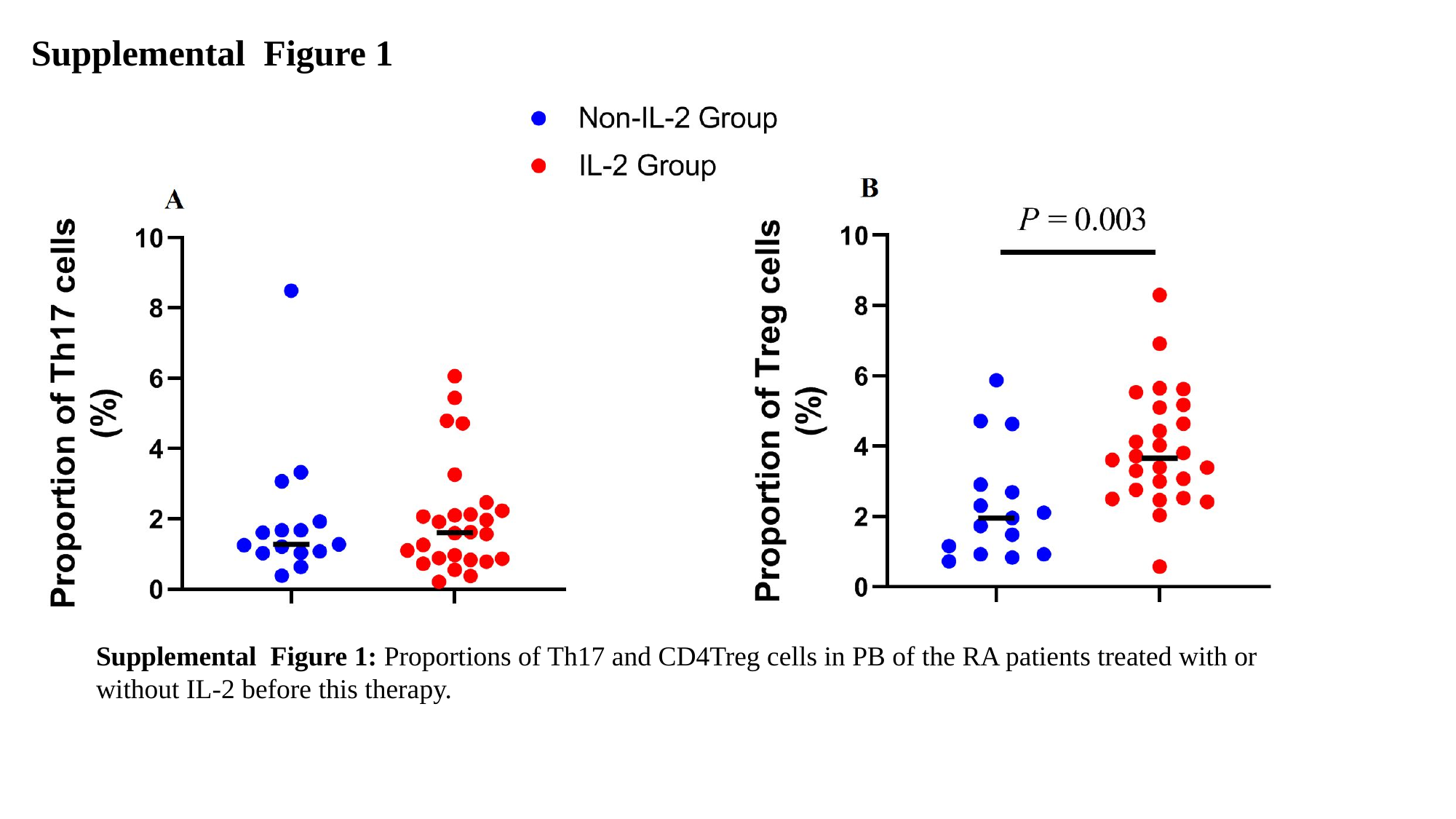

Supplemental Figure 1
Supplemental Figure 1: Proportions of Th17 and CD4Treg cells in PB of the RA patients treated with or without IL-2 before this therapy.

## Slide 2
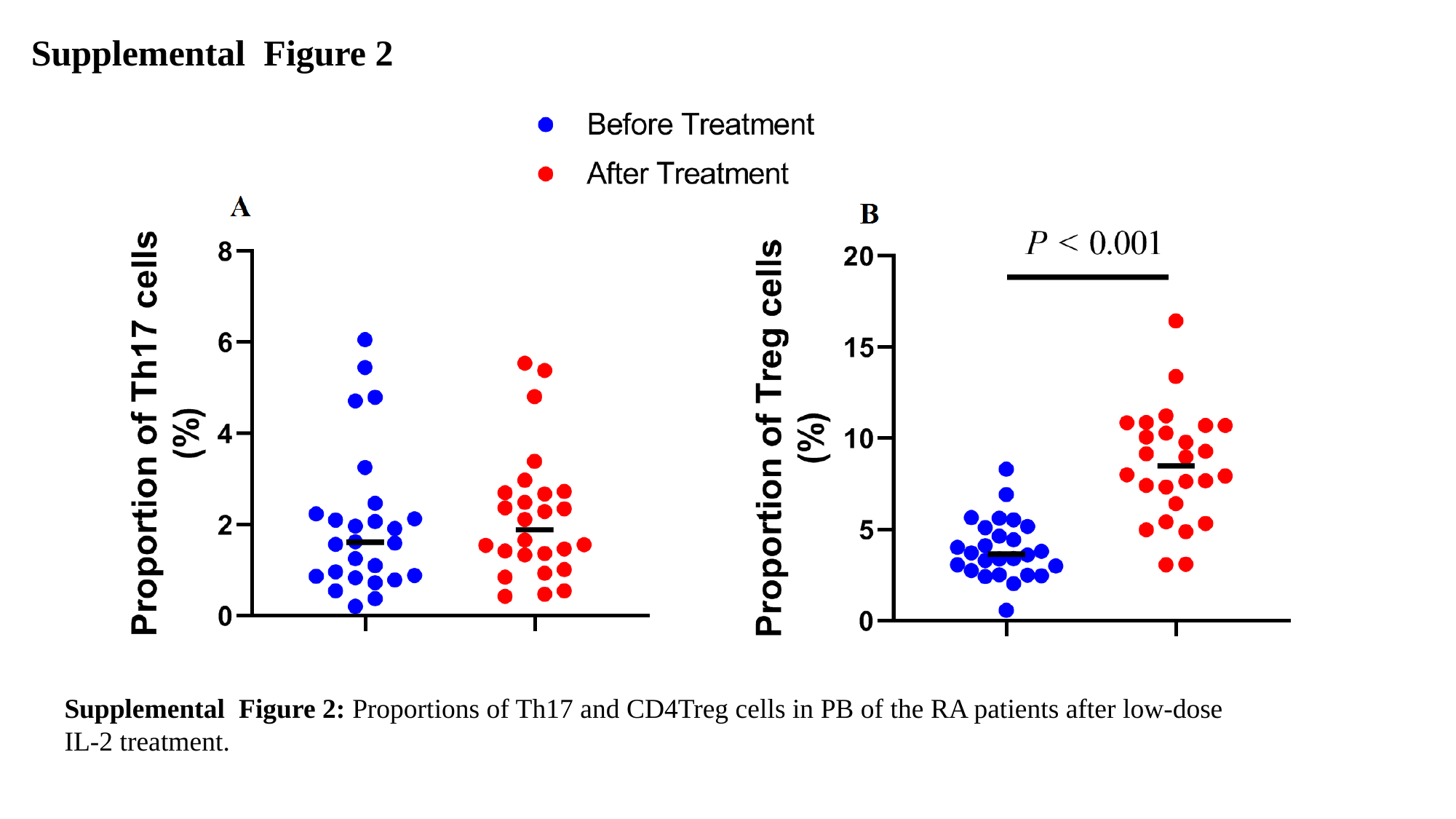

Supplemental Figure 2
Supplemental Figure 2: Proportions of Th17 and CD4Treg cells in PB of the RA patients after low-dose IL-2 treatment.

## Slide 3
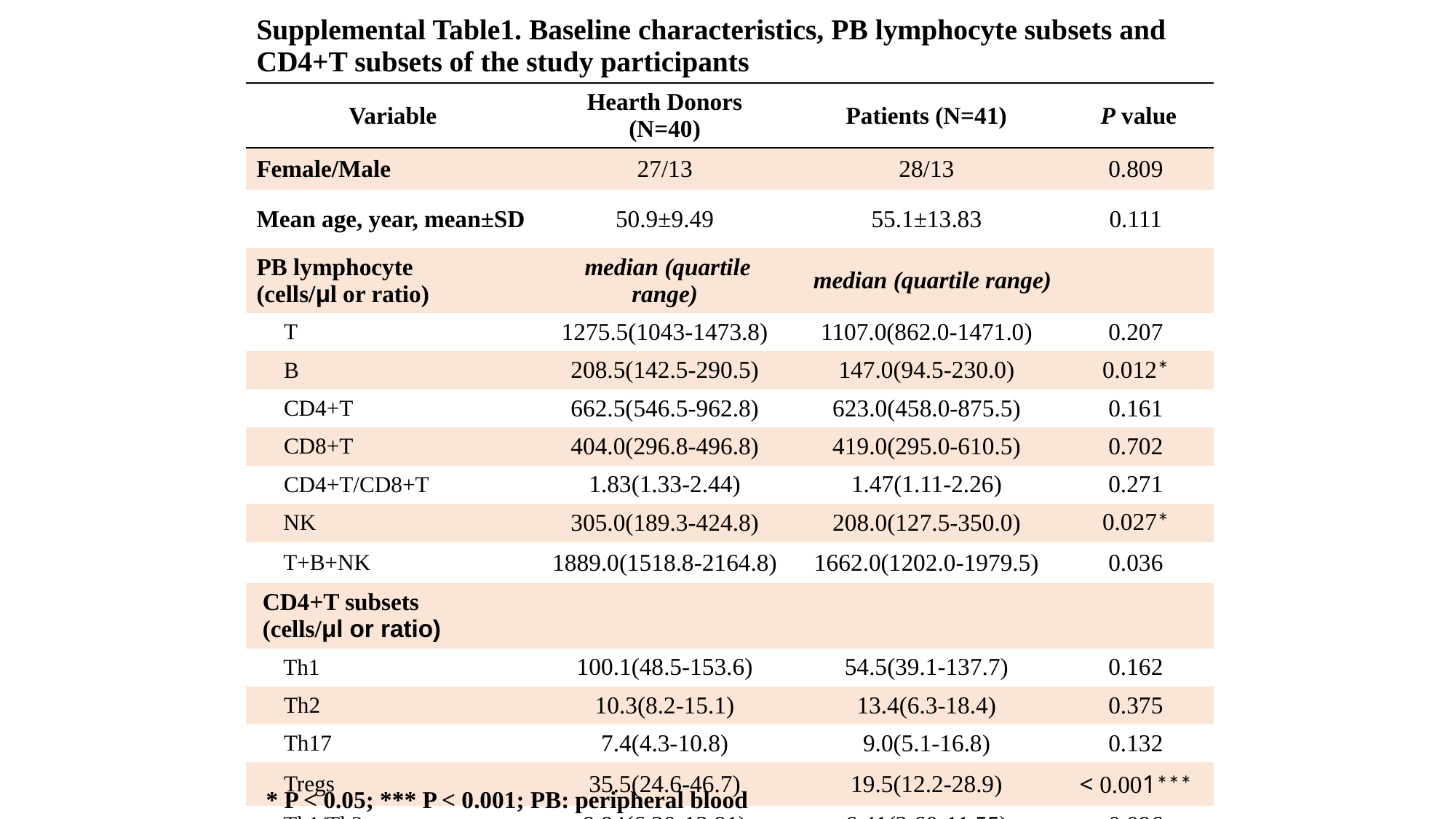

| Supplemental Table1. Baseline characteristics, PB lymphocyte subsets and CD4+T subsets of the study participants | | | |
| --- | --- | --- | --- |
| Variable | Hearth Donors (N=40) | Patients (N=41) | P value |
| Female/Male | 27/13 | 28/13 | 0.809 |
| Mean age, year, mean±SD | 50.9±9.49 | 55.1±13.83 | 0.111 |
| PB lymphocyte (cells/μl or ratio) | median (quartile range) | median (quartile range) | |
| T | 1275.5(1043-1473.8) | 1107.0(862.0-1471.0) | 0.207 |
| B | 208.5(142.5-290.5) | 147.0(94.5-230.0) | 0.012\* |
| CD4+T | 662.5(546.5-962.8) | 623.0(458.0-875.5) | 0.161 |
| CD8+T | 404.0(296.8-496.8) | 419.0(295.0-610.5) | 0.702 |
| CD4+T/CD8+T | 1.83(1.33-2.44) | 1.47(1.11-2.26) | 0.271 |
| NK | 305.0(189.3-424.8) | 208.0(127.5-350.0) | 0.027\* |
| T+B+NK | 1889.0(1518.8-2164.8) | 1662.0(1202.0-1979.5) | 0.036 |
| CD4+T subsets (cells/μl or ratio) | | | |
| Th1 | 100.1(48.5-153.6) | 54.5(39.1-137.7) | 0.162 |
| Th2 | 10.3(8.2-15.1) | 13.4(6.3-18.4) | 0.375 |
| Th17 | 7.4(4.3-10.8) | 9.0(5.1-16.8) | 0.132 |
| Tregs | 35.5(24.6-46.7) | 19.5(12.2-28.9) | < 0.001\*\*\* |
| Th1/Th2 | 9.94(6.20-13.91) | 6.41(2.60-11.55) | 0.096 |
| Th17/Tregs | 0.20(0.16-0.31) | 0.59(0.24-1.07) | < 0.001\*\*\* |
* P < 0.05; *** P < 0.001; PB: peripheral blood

## Slide 4
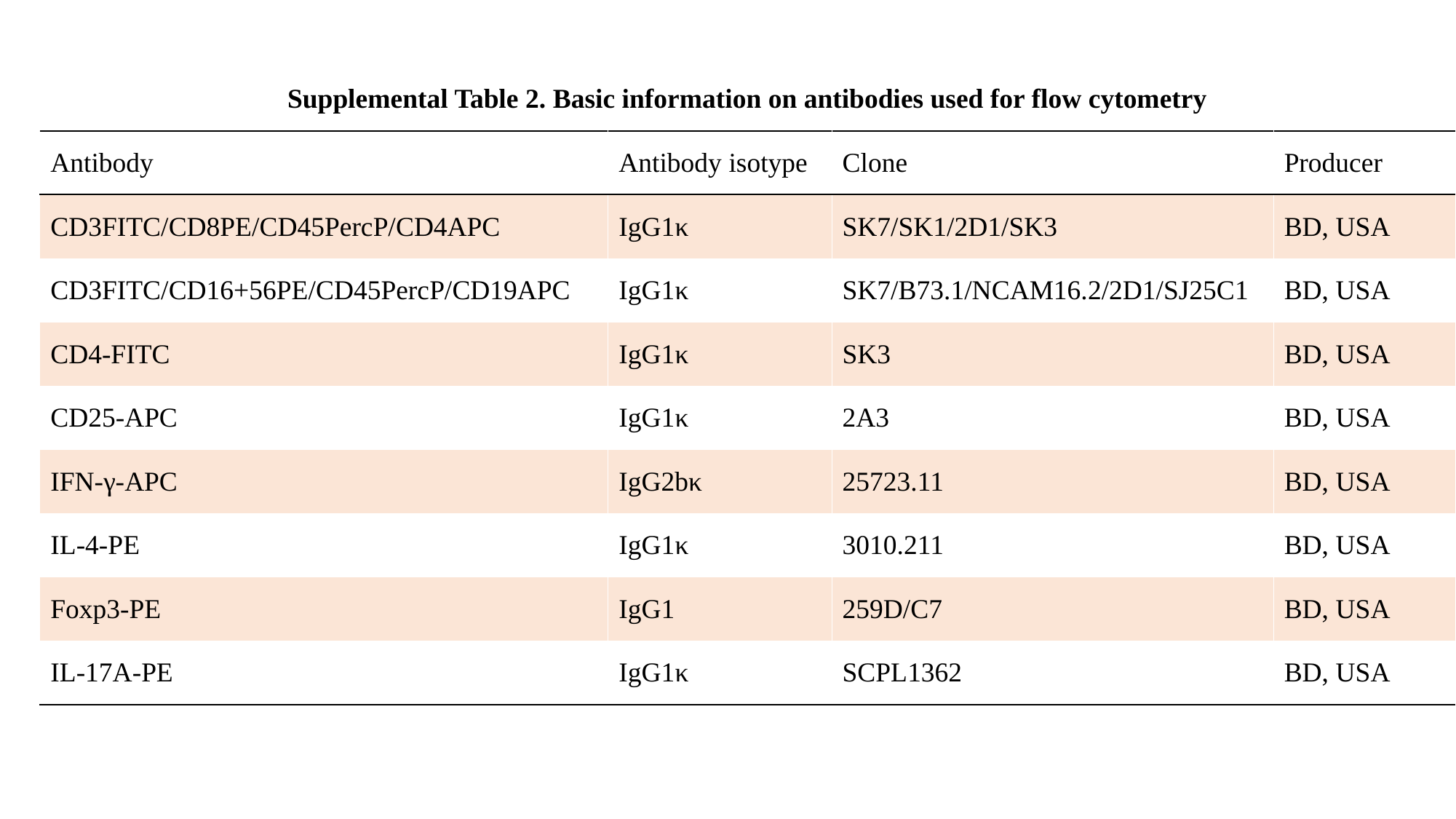

| Supplemental Table 2. Basic information on antibodies used for flow cytometry | | | |
| --- | --- | --- | --- |
| Antibody | Antibody isotype | Clone | Producer |
| CD3FITC/CD8PE/CD45PercP/CD4APC | IgG1κ | SK7/SK1/2D1/SK3 | BD, USA |
| CD3FITC/CD16+56PE/CD45PercP/CD19APC | IgG1κ | SK7/B73.1/NCAM16.2/2D1/SJ25C1 | BD, USA |
| CD4-FITC | IgG1κ | SK3 | BD, USA |
| CD25-APC | IgG1κ | 2A3 | BD, USA |
| IFN-γ-APC | IgG2bκ | 25723.11 | BD, USA |
| IL-4-PE | IgG1κ | 3010.211 | BD, USA |
| Foxp3-PE | IgG1 | 259D/C7 | BD, USA |
| IL-17A-PE | IgG1κ | SCPL1362 | BD, USA |

## Slide 5
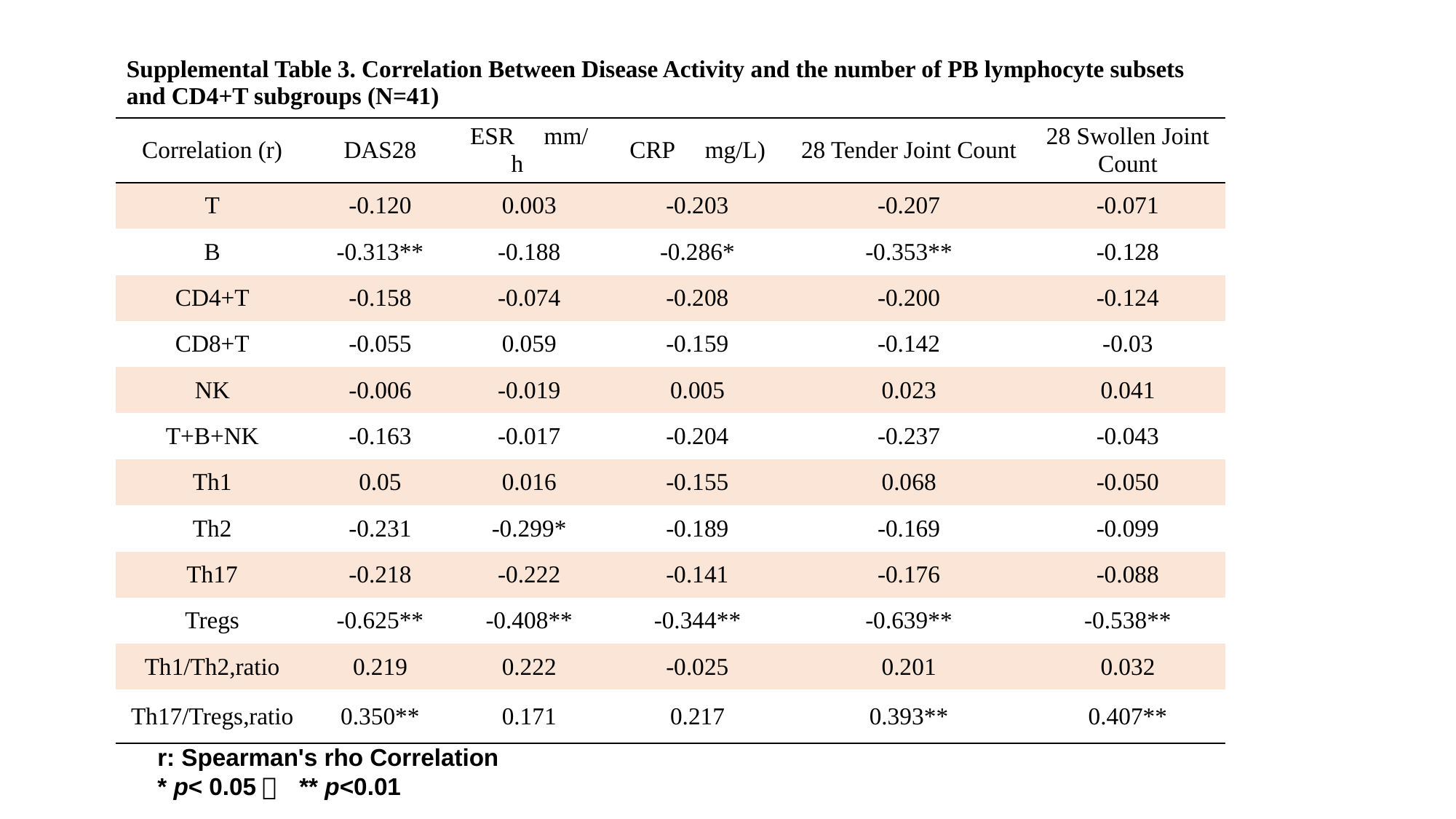

| Supplemental Table 3. Correlation Between Disease Activity and the number of PB lymphocyte subsets and CD4+T subgroups (N=41) | | | | | |
| --- | --- | --- | --- | --- | --- |
| Correlation (r) | DAS28 | ESR（mm/h） | CRP（mg/L) | 28 Tender Joint Count | 28 Swollen Joint Count |
| T | -0.120 | 0.003 | -0.203 | -0.207 | -0.071 |
| B | -0.313\*\* | -0.188 | -0.286\* | -0.353\*\* | -0.128 |
| CD4+T | -0.158 | -0.074 | -0.208 | -0.200 | -0.124 |
| CD8+T | -0.055 | 0.059 | -0.159 | -0.142 | -0.03 |
| NK | -0.006 | -0.019 | 0.005 | 0.023 | 0.041 |
| T+B+NK | -0.163 | -0.017 | -0.204 | -0.237 | -0.043 |
| Th1 | 0.05 | 0.016 | -0.155 | 0.068 | -0.050 |
| Th2 | -0.231 | -0.299\* | -0.189 | -0.169 | -0.099 |
| Th17 | -0.218 | -0.222 | -0.141 | -0.176 | -0.088 |
| Tregs | -0.625\*\* | -0.408\*\* | -0.344\*\* | -0.639\*\* | -0.538\*\* |
| Th1/Th2,ratio | 0.219 | 0.222 | -0.025 | 0.201 | 0.032 |
| Th17/Tregs,ratio | 0.350\*\* | 0.171 | 0.217 | 0.393\*\* | 0.407\*\* |
r: Spearman's rho Correlation
* p< 0.05； ** p<0.01

## Slide 6
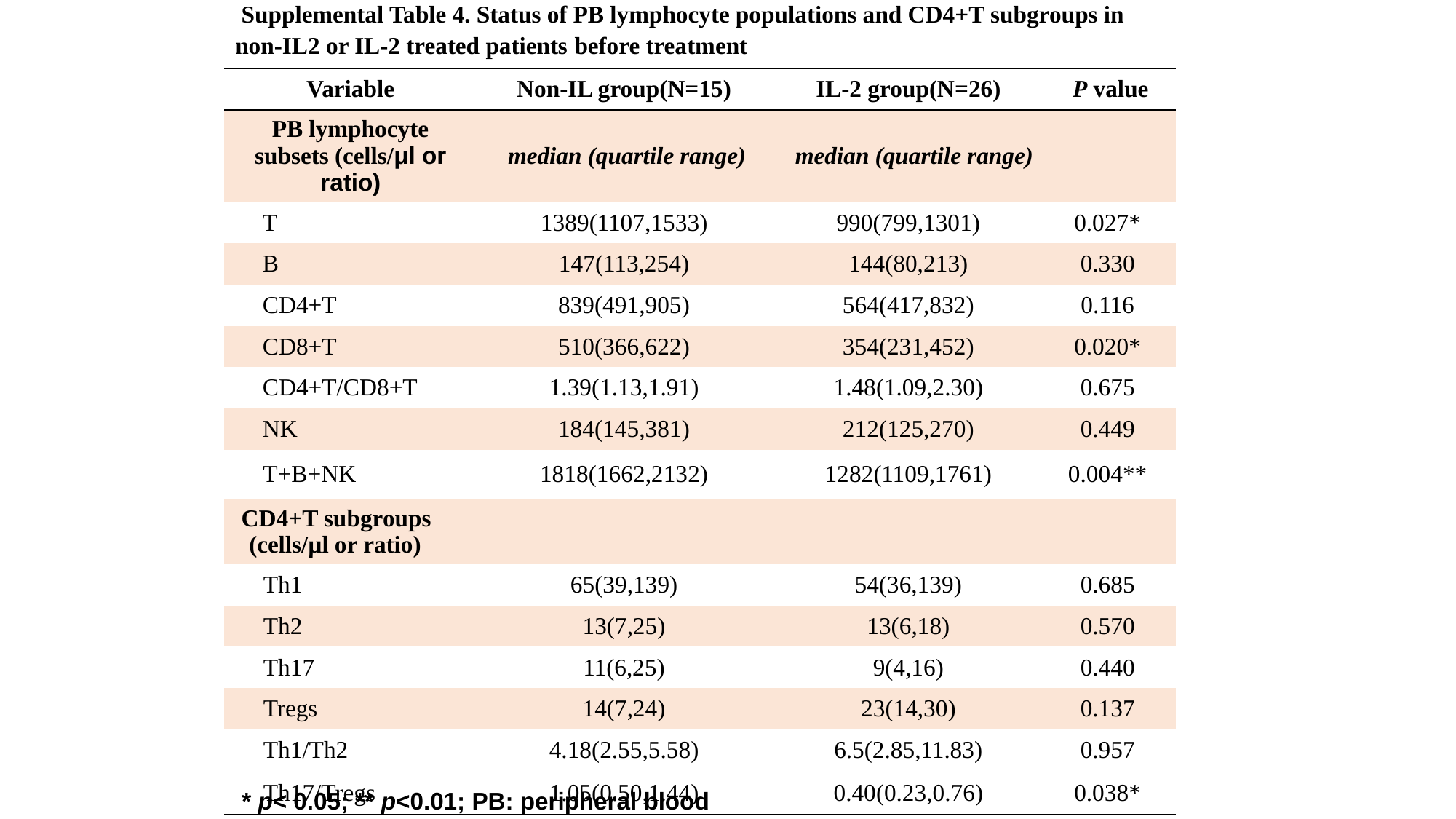

| Supplemental Table 4. Status of PB lymphocyte populations and CD4+T subgroups in non-IL2 or IL-2 treated patients before treatment | | | |
| --- | --- | --- | --- |
| Variable | Non-IL group(N=15) | IL-2 group(N=26) | P value |
| PB lymphocyte subsets (cells/μl or ratio) | median (quartile range) | median (quartile range) | |
| T | 1389(1107,1533) | 990(799,1301) | 0.027\* |
| B | 147(113,254) | 144(80,213) | 0.330 |
| CD4+T | 839(491,905) | 564(417,832) | 0.116 |
| CD8+T | 510(366,622) | 354(231,452) | 0.020\* |
| CD4+T/CD8+T | 1.39(1.13,1.91) | 1.48(1.09,2.30) | 0.675 |
| NK | 184(145,381) | 212(125,270) | 0.449 |
| T+B+NK | 1818(1662,2132) | 1282(1109,1761) | 0.004\*\* |
| CD4+T subgroups (cells/µl or ratio) | | | |
| Th1 | 65(39,139) | 54(36,139) | 0.685 |
| Th2 | 13(7,25) | 13(6,18) | 0.570 |
| Th17 | 11(6,25) | 9(4,16) | 0.440 |
| Tregs | 14(7,24) | 23(14,30) | 0.137 |
| Th1/Th2 | 4.18(2.55,5.58) | 6.5(2.85,11.83) | 0.957 |
| Th17/Tregs | 1.05(0.50,1.44) | 0.40(0.23,0.76) | 0.038\* |
 * p< 0.05; ** p<0.01; PB: peripheral blood
